# Broad inhibition of *plasmodium falciparum *cytoadherence by (+)-epigallocatechin gallate

**DOI:** 10.1186/1475-2875-10-348

**Published:** 2011-12-01

**Authors:** Pradeep R Patil, Sandra Gemma, Giuseppe Campiani, Alister G Craig

**Affiliations:** 1 European Research Centre for Drug Discovery and Development, Department of Pharmaceutical and Applied Chemistry, University of Siena, via Aldo Moro 2, Siena 53100, Italy; 2 Liverpool School of Tropical Medicine, Pembroke Place, Liverpool L3 5QA, UK; 3 Université Montpellier II Sciences et Techniques, Montpellier, France

**Keywords:** Plasmodium falciparum, Malaria, Cytoadherence, Inhibitor, ICAM-1

## Abstract

**Background:**

The surface antigen P*f*EMP-1 is a key virulence factor of the human malaria parasite implicated in the cytoadherence of *Plasmodium falciparum *infected erythrocytes to a range of receptors on host endothelium. Among these host receptors, binding to ICAM-1 is related to cerebral malaria. The majority of the mortality in children with cerebral malaria is seen within 24 h of hospital admission despite the use of effective anti-parasite drugs, therefore, the development of adjunctive therapies is urgently needed.

The polyphenolic compound (+)-epigallocatechin gallate ((+)-EGCG) has been previously evaluated for anti-adhesive properties using a small number of laboratory parasite isolates. Here, this property is further explored using a new panel of ICAM-1-binding patient isolates of *P. falciparum *to ascertain if (+)-EGCG might be effective as a broad spectrum inhibitor of ICAM-1-based cytoadherence.

**Methods:**

*Plasmodium falciparum *lines, including A4 and ItG as positive controls and nine new ICAM-1 binding patient isolates, were allowed to bind with ICAM-1-Fc protein under static assay conditions in the presence and absence of 50 μM (+)-EGCG. Adhesion levels of all the parasite strains were quantified by microscopy as the mean number of infected erythrocyte (IE) bound per mm^2 ^of surface area and statistical comparisons were made to demonstrate the effect of (+)-EGCG on the binding of various parasite variants to human ICAM-1.

**Results:**

This study revealed that binding of patient isolates to ICAM-1 was reduced significantly with inhibition levels of 37% in patient isolate BC-12 up to a maximum of 80% in patient isolate 8146 at 50 μM (+)-EGCG.

**Conclusion:**

Evaluation of the anti-adhesive property of (+)-EGCG against a new panel of ICAM-1-binding patient isolates of *P. falciparum *showed that this inhibitor, identified as potential mimic of the L43 loop of human ICAM-1, was effective at blocking cytoadherence.

## Background

*Plasmodium falciparum *malaria remains a major life-threatening parasitic disease, killing approximately 1 million people each year worldwide, mainly in sub-Saharan Africa [1]. Children and pregnant women are most susceptible. This infection can progress unpredictably to severe forms, including anaemia and cerebral malaria. The surface antigen P*f*EMP-1 (*P. falciparum *erythrocyte membrane protein-1) is a key virulence factor of the human malaria parasite, encoded by about 60 *var *genes per haploid genome [2]. P*f*EMP-1 variants have been implicated in the cytoadherence of *P. falciparum*-infected erythrocytes (IEs) to several binding receptors on host vascular endothelium [3-5], sequestering infected cells away from the spleen, which would otherwise destroy them. Switching of *var *gene expression allows the parasite to modify the antigenic and functional properties of IEs, thereby evading immunity and affecting infection outcome. In most cases the parasite expresses a single *var *gene at a time, maintaining all other members of the family in a transcriptionally silent state [6].

Among the many host receptors, binding to Intercellular Adhesion Molecule -1 (ICAM-1) has been shown to be related to cerebral malaria [7]. The majority of the mortality with cerebral malaria is seen within 24 h of hospital admission despite the use of effective anti-parasite drugs [8], therefore, the development of adjunctive therapies is urgently needed. The polyphenolic compound (+)-epigallocatechin gallate ((+)-EGCG) has been shown to have anti-adhesive properties [9]. This compound was identified using *in silico *molecular alignment techniques based on DE loop of human ICAM-1, which is a common feature of the ICAM-1 binding sites for at least the three *P. falciparum *laboratory variants tested [10]. (+)-EGCG inhibited binding of two variant ICAM-1 binding parasite lines at micromolar concentrations in a highly specific, dose-dependent manner. Interestingly, binding to CD36 was partially inhibited by EGCG in an ICAM-1-binding line, but not in a laboratory isolate that showed no binding to ICAM-1. In this current study the anti-adhesive properties of (+)-EGCG were measured against a new panel of ICAM-1-binding patient isolates of *P. falciparum *in order to evaluate the breadth of the cytoadherence inhibitory effect.

## Methods

### Recombinant protein and cytoadherence inhibitor

(+)-EGCG, which is known to show anti-cytoadherence properties with A4 and ItG parasite strains, was used at 50 μM concentration. The purified receptor protein used in this study was human ICAM-1-Fc, which was prepared as previously described [11].

### ICAM-1 selection of parasite lines using Dynabeads

50 μl Protein A Dynabeads (Invitrogen) were washed 3 times with 500 μl PBS/1% BSA and then resuspended in 100 μl PBS/1%BSA. 2.5 μg ICAM-1-Fc protein was added to the bead suspension and made up to 400 μl by adding PBS/1%BSA. This was then incubated at room temperature by rotating for 1 h, again washed with 500 μl PBS/1% BSA using a magnet to retain the beads each time. Beads were resuspended in 200 μl PBS/1% BSA and stored at 4°C overnight.

Parasite culture was enriched for trophozoite stages using Plasmion by standard protocols [12]. 50 μl of Plasmion enriched parasites and 150 μl PBS/1% BSA were added to previously prepared ICAM-1 labelled Dynabeads. The mixture was incubated at room temperature, rotating for 45 min then gently washed twice with 500 μl PBS/1% BSA using a magnet to retain the beads each time. Beads were resuspended in parasite media and cultured with fresh washed red blood cells (RBCs).

### Parasite culture

*Plasmodium falciparum *lines used in this study were the laboratory lines A4 and ItG, as a positive controls, and patient isolates PO-69, PCM-7, GL-6, BC-31, BC-12, 8206, 8146, 8131, 6392, which have previously been selected for binding to ICAM-1. Separate experiments had shown, using mutant ICAM-1 proteins, that all the ICAM-1-binding isolates used in this study have the L43 loop of ICAM-1 as a critical part of their binding site. To minimize the effect of antigenic switching in vitro the selected parasite lines were maintained in culture for not more than 3 weeks post-selection. RBCs for parasite culture were purified away from mononuclear cells and granulocytes using Lymphoprep.

Parasites were grown to maturity for 24-48 h from frozen stabilates. Laboratory parasite lines and parasites from patient isolates were cultured at 1% haematocrit in group O human erythrocytes using standard culturing techniques, using RPMI 1640 medium (supplemented with 37.5 mM HEPES, 7 mM D-glucose, 6 mM NaOH, 40 μg gentamicin sulfate/ml, 2 mM L-glutamine, and 10% human serum) at a pH of 7.2 in a gas mixture of 96% nitrogen, 3% carbon dioxide, and 1% oxygen. The parasitaemia was calculated by determination of the number of IEs per 500 red blood cells (RBC) for thin blood films. Baseline RBC counts were used to calculate the parasite density (parasites/μl). For all adhesion assays the IE suspension was adjusted to 3% parasitaemia and 1% haematocrit.

### Static adhesion assays

Purified recombinant ICAM-1-Fc protein and PBS only (as negative control) were spotted in triplicate in a radial pattern using 2 μl spots on 60 × 50 mm bacteriological plastic Petri dishes (Falcon 1007; Becton Dickinson, Oxford, UK) at concentrations of 50 μg/ml for ICAM-1. This concentration had previously been shown to be within the dynamic range for detecting differences in adhesion and produce coated surfaces with receptors at levels approximately equal to receptor densities seen on activated endothelium [13]. The dishes were placed in a humidified chamber for 2 h at 37°C to allow the proteins to adsorb to the surface of Petri dish, after which the protein solution and PBS were aspirated off and the uncoated plastic area was blocked overnight with 1% BSA/PBS at 4°C. The plates were warmed at 37°C for one hour, blocking solution (1% BSA/PBS) was removed and plates were washed with binding buffer (RPMI 1640 with 0.2% glucose) prior to adding 1.5 ml of parasite culture (3% parasitaemia; 1% haematocrit in binding medium), with and without 50 μM (+)-EGCG. The plates were incubated at 37°C for one hour with gentle resuspension every 10 min. Unbound infected and uninfected erythrocytes were removed by gentle manual washing (4-6 washes) with 2 ml binding medium per wash (monitoring of adhered cells was performed using an inverted microscope). The adhered IEs were fixed with 1% glutaraldehyde in phosphate buffered saline for 1 h and stained with 10% Giemsa for 30 min. Adhesion levels of all parasite strains (with and without (+)-EGCG) were quantified by microscopy using a unique, anonymous identifier for each dish (with the operator blinded to the IE category) and results were expressed as the mean number of IEs bound per mm^2 ^of surface area. Inhibition experiments were conducted twice for isolates PO-69, 8206 and 8131, and three times for the other parasite lines. The results were compared to quantify the effect of (+)-EGCG on binding of various ICAM-1-binding patient isolates to human ICAM-1.

## Results and discussion

**Figure 1:**
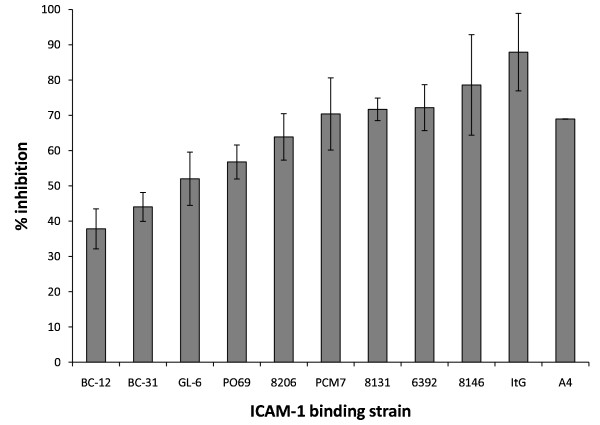
**Screening of (+)-EGCG against new ICAM-1 binding patient isolates of *P. falciparum***. EGCG was used at 50 μM concentration during the binding assays. Results are shown as mean inhibition (compared to no inhibitor control) ± standard deviation.

This study focussed on the ability of the naturally occurring polyphenolic compound (+)-EGCG to inhibit the binding of various patient isolates to ICAM-1. All the isolates tested showed significantly reduced adhesion with inhibition ranging from 37%-80% (Figure 1). This varying degree of inhibition of various patient isolates by (+)-EGCG, may refer to variable contact residues on P*f*EMP-1 of different patient isolates involved in the binding with ICAM-1 as seen in previous studies [10].

On the basis of these results it can be concluded that, despite the variation in the contact residues engaged on the BED side of ICAM-1 by different P*f*EMP-1 variants [14], some structural similarity is retained. It seems likely that the L-43 loop of human ICAM-1 is a key domain in the interaction with P*f*EMP-1 based on the observation that the whole set of ICAM-1 binding patient isolates was inhibited by (+)-EGCG, which is known to mimic L-43 loop of ICAM-1. Although several other binding activities use the N-terminal domain of ICAM-1 (e.g. T-cells, rhinovirus, fibrinogen), the L43 loop appears to be unique to *P. falciparum*-infected erythrocytes, supporting its use as a starting point for the design of specific inhibitors of cytoadherence. However, there are still a number of issues that still need to be resolved before taking (+)-EGCG further, such as its ability to reverse existing cytoadherence rather than just inhibition of new binding and testing the compound for disruption of ICAM-1-dependent IE binding in the context of endothelial cells. Also, to improve the pharmacological characteristics of (+)-EGCG, it is necessary to 'remake' the chemical scaffold to produce a lead structure that is more amenable to chemical synthesis. These experiments are underway.

## Conclusions

(+)-EGCG is able to inhibit binding to ICAM-1 by a range of patient-derived parasite isolates. This supports further development of anti-adhesive compounds with broad reactivity based on this chemical scaffold as novel adjunct therapies for cerebral malaria.

## List of abbreviations

BSA: Bovine Serum Albumen; EGCG: Epigallocatechin gallate; ICAM-1: Intercellular Adhesion Molecule - 1; IE: Infected erythrocyte; PBS: Phosphate buffered saline; PfEMP-1: Plasmodium falciparum erythrocyte membrane protein - 1; RBC: Red blood cell

## Competing interests

The authors declare that they have no competing interests.

## Authors' contributions

SG, GC & AC designed the study; PRP carried out the experimental work; PRP & AC wrote the manuscript; SG & GC reviewed the final draft. All authors read and approved the final manuscript.
